# Optimization on theBuried Depth of Subsurface Drainage under Greenhouse Condition Based on Entropy Evaluation Method

**DOI:** 10.3390/e20110859

**Published:** 2018-11-08

**Authors:** Maomao Hou, Zhiyuan Lin, Jingnan Chen, Yaming Zhai, Qiu Jin, Fenglin Zhong

**Affiliations:** 1 College of Horticulture, Fujian Agriculture and Forestry University, Fuzhou 350002, China; 2 College of Horticulture and Landscape Architecture, Fujian Vocational College of Agriculture, Fuzhou 350002, China; 3 College of Water Conservancy and Hydropower, Hohai University, Nanjing 210098, China; 4 State Key Laboratory of Hydrology-Water Resources and Hydraulic Engineering, Nanjing Hydraulic Research Institute, Nanjing 210098, China

**Keywords:** entropy weight, subsurface drainage, tomato, multi-index, evaluation

## Abstract

Numerous indicators under the plant-soil system should be taken into consideration when developing an appropriate agricultural water conservancy project. Entropy evaluation method offers excellent prospects in optimizing agricultural management schemes. To investigate the impact of different buried depths (30, 45, 60, 75, 90, and 105 cm) of subsurface drainage pipes on greenhouse plant-soil systems, the tomato was employed as plant material, and the marketable yield, fruit sugar to acid ratio, soil electrical conductivity, nitrogen loss rate, as well as crop water and fertilizer use efficiency were observed. Based on these indicators, the entropy evaluation method was used to select the optimal buried depth of subsurface drainage pipes. Both the calculation results of objective and subjective weights indicated that tomato yield and soil electrical conductivity were relatively more crucial than other indexes, and their comprehensive weights were 0.43 and 0.34, respectively. The 45 cm buried depth possessed the optimal comprehensive benefits, with entropy evaluation value of 0.94. Under 45 cm buried depth, the loss rate of soil available nitrogen was 13.9%, the decrease rate of soil salinity was 49.2%, and the tomato yield, sugar to acid ratio, nitrogen use efficiency, and water use efficiency were 112 kg·ha^−1^, 8.3, 39.7%, and 42.0%, respectively.

## 1. Introduction

The productivity of irrigated agriculture is being seriously affected by waterlogging disasters, which are mainly caused by increasing water consumption, low water use efficiency, and poor natural drainage conditions. At present, there are three common methods being used worldwide: Ditch drainage, subsurface drainage, and vertical well drainage [1]. Subsurface drainage is superior to ditch drainage in lowering groundwater level, and the subsurface pipe occupies less space, which is propitious to mechanized operations. Therefore, introducing and promoting new technology, such as subsurface drainage can play an important role in the sustainable development of irrigation agriculture, and is of great importance in improving soil ecological environment, regulating the status of water, fertilizer, gas, and heat in soil, thereby increasing crop productivity, and expanding the area of cultivated land [2]. 

The subsurface drainage firstly appeared in the United Kingdom and then was widely adopted by the United States, the Soviet Union, Japan, the Netherlands, Czechoslovakia, and Poland. Originally, subsurface drainage was used to lower the ground water level. Until now, subsurface drainage is effective in both water and salt elimination. In China, subsurface drainage has been widely used in coastal water-logging areas and shallow saline-alkali areas in Zhejiang, Shandong, Xinjiang, and Ningxia [3,4].

Salt reduction using subsurface drainage follows the regularity of "salt comes along with water and salt goes with water". The fully dissolved salt in soil is penetrated into the deep soil layer then discharged through the drain pipe, therefore the soil salt content is effectively reduced, and the groundwater level is controlled, meanwhile the soil physical and chemical properties are improved. Shallow subsurface drainage in Xinjiang has proved to reduce soil salinity, increase cotton output, and leach ions such as Cl^−^, Na^+^, and SO_4_^2−^ [5]. The management of subsurface drainage can change the status of farmland sludge caused by waterlogging, enhance the activity of soil microorganisms, promote the deep rooting of crop roots, facilitate the absorption of deep soil nutrients, and accelerate the growth process of crops. After the surface water in the farmland is quickly eliminated, subsurface drainage is manifested in: (1) Improving the aeration of the soil and increasing the earth temperature; (2) strengthening the soil microbial activity and improving the fertilization effect; and (3) promoting crop root growth and increasing the yield [6].

The layout scheme of the subsurface drainage pipe is determined by various indicators [7], generally, pre-experiments should be performed in the early stage of agricultural water conservancy construction. In the pre-experiment, several indicators under different layout schemes of subsurface drainage, such as the crop yield, quality, soil salinity and nutrient distribution, and groundwater level, are usually observed. Owing to a great number of indicators and schemes, the optimization of subsurface drainage schemes will be transformed to a decision-making issue with multi-objectives and multi-indexes. When subjectively incapable of judging the optimal layout scheme of drainage pipes, it is necessary to draw support from statistical means, such as the entropy weight method, the projection pursuit method, as well as the principal component analysis method.

The basic principle of the entropy method is that a greater degree of difference in the index value will produce smaller information entropy, and this index will have a greater weight and provide more information. Conversely, a smaller degree of difference in the index value produces greater information entropy, and this index has a smaller weight and provides less information. The entropy weight method is based on real data from the project, therefore, the calculation result will bemore objective, and the decision result is more reliable. Given its simplification, the entropy method is widely used to solve multi-objective decision-making issues [8,9].

Our early experiments indicated that subsurface drainage played an important role in improving greenhouse saline soils. However, since the early experiments contained only two levels of buried depth of drainage pipe, the depth should be further calibrated. In this study, we employed the entropy weight method to evaluate the comprehensive effects of different buried depths of subsurface drainage. The objective of this study was to: (1) Find out the optimal buried depth of drainage pipes, for providing a theoretical basis for local practice in scheduling the layout scheme of subsurface drainage; and (2) evaluate the effects of entropy method in developing agricultural water management schemes, to provide references for other similar projects.

## 2. Materials and Methods 

### 2.1. Experimental Site

The experiments were conducted from May to October of 2017 in Changle, Fuzhou city, Fujian province of China. Changle belongs to a mid-subtropical marine monsoon climate zone, which is warm and humid. The summer duration is long without much heat, and the winter is short with less frost and snow. The average annual temperature from 2005 to 2015 was 19.3 °C. In January, the average temperature was 10.3 °C, and the extreme low temperature was −1.3 °C. The average temperature in July was 28.3 °C, and the extreme high temperature was 37.4 °C. The frost free period is 333 days. The average rainfall is 1382.3 mm. The average annual wind speed is 4.1 m/s, and most are northeast winds. The soils used for the experiment were collected from the greenhouse, which has continuously been cultivated for tomatoes for 8 years. The soils were seriously secondary salinized. Soils in plough layer (0–20 cm) were characterized by electrical conductivity of 4.51 ms·cm^−1^, pH of 5.6, bulk density of 1.41 g·cm^3^, total porosity of 46.2%, available N content of 180 mg·kg^−1^, available P of 18.1 mg·kg^−1^, and available K of 174 mg·kg^−1^.

### 2.2. Experimental Design

**Figure 1:**
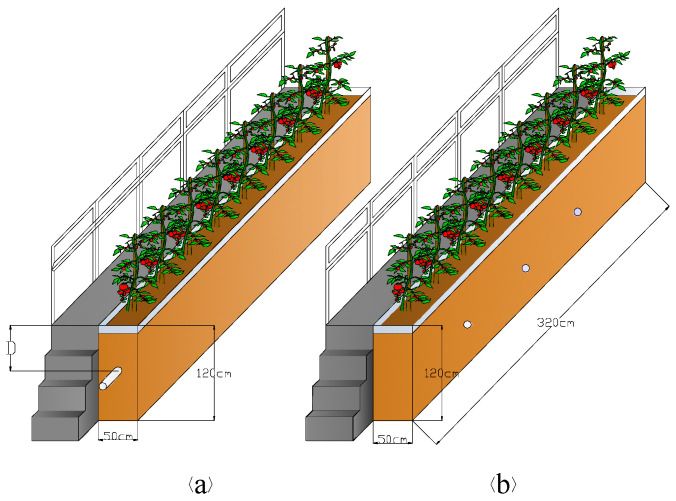
Experimental device with subsurface drainage (**a**) and without subsurface drainage (**b**).

The experiment was carried out in the device shown in Figure 1. The main material of the device was transparent resin. The device was 120 cm in height, 50 cm in width, and 320 cm in length. On one side of the device, there were staircase trails for observing soil-plant indicators. Armrests beside the footpath ensured the safety of observers. Six buried depths of 30 cm (D1), 45 cm (D2), 60 cm (D3), 75 cm (D4), 90 cm (D5), and 105 cm (D6) were arranged. The buried depth was the distance from the soil surface to the center of the pipe. The pipe was a porous PVC bellow with a diameter of 10 cm and a slope of 1/600. Non-woven fabric was wrapped around the pipe to prevent pore blockage. The design of the device is shown in Figure 1a. Our experiment also employed a control treatment shown in Figure 1b. For the control treatment, no pipe was buried in the soil, but three drainage holes were opened beside the device to discharge excessive water when waterlogging occurs. However, no drainage holes were used from the beginning to the end of the experiment.

The tomato variety “Yingshidahong” was used as the plant material. Their seedlings were cultivated in 15 × 10 seedling-raising plates. The seedlings were transplanted when they had six expanded leaves. The transplant date was May 18. The lower limits of soil water content were controlled at 60%, 70% and 70% (accounted for the field capacity), respectively, for the seedling stage, fruiting stage, and harvest stage, and the upper limits were all at 90%. Once the soil moisture content had fallen to the lower limit, irrigation was started. The water supply of irrigation was manual. After planting, conventional management was carried out in the field. The fertilizer application and the irrigation regime were the same amongst the different treatments. The ^15^N-labeled urea (abundance of 10.28%) was used as nitrogen fertilizer, and the N application amount was 120 kg/ha. Calcium guolinate and potassium sulfate were used as phosphate and potassium fertilizer, and the application amount of N:P:K_2_O was 1:1:2. Additionally, 50% of the fertilizers were applied before the first panicle. The plant was pinched after reserving 3 head sprouts. Other field management was consistent with local practice. 

### 2.3. Measurement

The indicators used in this study to evaluate the effects of subsurface drainage schemes included the tomato marketable yield, sugar-acid ratio, nitrogen use efficiency, water use efficiency, salt reduction rate, and available nitrogen loss rate. The ratio of sugar to acid determines the taste of tomato fruit. The marketable tomato yield is calculated using the total yield minus the yield of diseased and malformed fruit. 

For each treatment, 6 mature fruits were randomly collected, and their pulps were mixed before being tested. The total sugar was measured using the Fehling reagent titration method and the total acid was measured using the sodium hydroxide titration method, as described in Reference [10].

At harvest stage, plant samples were collected and then separated by root, stem, leaf, and fruit. The plant organs were placed into an oven. After being killed for half an hour at 105 °C, the tomatoes were dried at 75 °C. The dried organs were sifted and grinded to measure the ^15^N abundance. The abundance of ^15^N was measured through mass spectrometry (Finniga-Mat-251, Mass-Spectrometers, Finnigan, Germany) at Nanjing Institute of Soil Research, Chinese Academy of Sciences. The nitrogen use efficiency was calculated using tracing technology as in Reference [11].

Water use efficiency was calculated using the water balance method, as outlined in Reference [12].

Soil electrical conductivity was measured each month from May 18 to September 18 using a soil conductivity meter (delta-t, Cambridge, UK). The salt reduction rate was calculated using the electrical conductivity values on May 18 and September 18.

The available N concentration was determined by the alkali solution diffusion method, as in Reference [13].

### 2.4. Entropy Weight Method for Optimization

The modeling of the entropy method followed Reference [14]:

Supposing that there are *n* evaluation indexes and *m* different buried depths of drainage pipes, *m* depths combined with *n* indexes can form a matrix:

$$R={\left(r_{ij}\right)}_{m\times n}$$

where; *r_ij_* is the *jth* evaluation index of the *ith* depth. For *r_j_*, there is information entropy (average amount of information after excluding redundancy):

$$E_{j}=-\sum_{i=1}^{m}p_{ij}\ln p_{ij},\left(j=1,2,3,\ldots ,n\right)$$

*p_ij_* is calculated from the formula:

$$p_{ij}=r_{ij}/\sum_{i-1}^{m}r_{ij}$$

The entropy value of *jth* index:

$$e_{j}=\frac{1}{\ln m}E_{j},\;\left(j=1,\;2,\;3,\ldots ,\;n\right)\;$$

The objective weight of *jth* index:

$$\theta_{j}=\left(1-e_{j}\right)/\sum_{i=1}^{n}(1-e_{j}),\;\left(j=1,\;2,\;3,\;\ldots ,\;n\right)\;$$

It is clear that:

$$0\leq \theta_{j}\leq 1;\sum_{j=1}^{n}\theta_{j}=1$$

Comprehensive index weight can be obtained by combining subjective weights *w_1_, w_2_, w_3_, …, w_n_* (given by decisionmaker) with objective weight *θ_j_* (*j* = 1, 2, 3, …, *n*):

$$\alpha_{j}=\theta_{j}\bar{\omega_{j}}/\sum_{j=1}^{n}\theta_{j}\bar{\omega_{j}},\;\left(j=1,\;2,\;3,\ldots ,\;n\right)$$

The optimum value of each row was recorded as r_j_*, and the index value in the matrix (*R*) was normalized. The indexes can be divided into two classes, namely the profitable index and the damage index. Regarding the profitable index (marketable yield, sugar to acid ratio, water and nitrogen use efficiency, available N content), the index value was expected to be higher. While for the damage index (soil electrical conductivity), the index value was expected to be lower. The normalization methods for the profitable and damage index are:

$$d_{ij}=\left\{ \begin{array}{l} \frac{r_{ij}}{r_{j}{}^{*}},\;r_{j}{}^{*}=\max\left\{r_{ij}\right\}\\ \frac{r_{j}{}^{*}}{r_{ij}},\;r_{j}{}^{*}=\min\left\{r_{ij}\right\} \end{array}\right.$$

The entropy weight evaluation value (i.e., the optimal scheme that will obtain the highest entropy weight evaluation value) under each buried depth can be calculated from:

$$\lambda_{i}=\sum_{j=1}^{n}\alpha d_{ij},\;i=1,\;2,\;3,\ldots ,\;m.\;$$

### 2.5. Statistic Analysis

Data were input to SPSS 18.0 software to compare the difference according to Duncan’s multiple range test, as explained in Reference [15].

## 3. Results

### 3.1. Changes of Soil Salinity and Available Nitrogen Content

**Figure 2:**
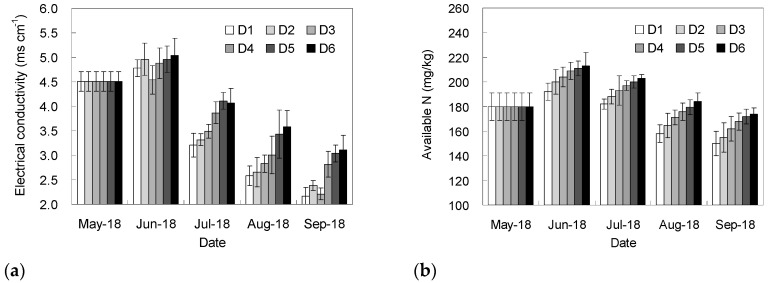
The dynamics of soil electrical conductivity (**a**) and available nitrogen content (**b**) (D1, D2, D3, D4, D5, and D6 represent 30, 45, 60, 75, 90, and 105 cm buried depths of subsurface drainage pipes, respectively).

Figure 2 shows the change of soil electrical conductivity and available nitrogen content in the plough layer. The electrical conductivity and available nitrogen content firstly increased, then decreased. Owing to fertilizer application, from May 18 to June 18, the content of available nitrogen increased. The fertilizers also brought in salt-based ions, resulting in the increase in electrical conductivity. After June 18, as the irrigation amount increased, the total water amount in the soil increased, therefore the drainage became more fluent, and the drainage by the buried pipes began to be effective. On September 18, the conductivity and available nitrogen content in all the treatments reached the lowest values, and the overall performance was that a shallower buried depth led to a lower conductivity and available nitrogen content in the soil, indicating that shallow a buried pipe was conducive to a rapid reduction of soil salt, but would cause a greater loss of available nitrogen. At the end of the experiment, the conductivity of each treatment decreased by 31.0%–51.9%, and the decrease rate of D1 was the highest. The available nitrogen contents in the treatments decreased by 3.3%–16.7%, and the lowest loss rate of available nitrogen was detected in D6. The available nitrogen content basically reached equilibrium before and after the experiment, probably due to the supply of fertilizer. 

### 3.2. Yield, Sugar to Acid Ratio, Nitrogen, and Water Use Efficiency

Table 1 shows the tomato marketable yield, sugar to acid ratio, nitrogen use efficiency, and water use efficiency under different treatments. There was no significant correlation between yield and burial depth. D2 obtained the highest yield of 112.0 kg ha^-1^, which was significantly (*p* < 0.05) higher than other treatments, whilst the yield under D3 was the lowest (86.3 kg·ha^-1^). The sugar to acid ratio was positively correlated with buried depth. D6 achieved the highest sugar to acid ratio of 9.90, whilst the lowest was in D1, and was recorded as 7.73. Significant difference of sugar to acid ratio was found between D6 and D1. The highest nitrogen use efficiency and water use efficiency were detected in D6 and D1, and were 49.5% and 45.0%, respectively. It was also discovered that deeper buried depths of drainage pipes would result in higher nitrogen use efficiency.

### 3.3. Optimization of Subsurface Drainage Schemes

The evaluation index system was established based on the six indicators in Figure 2 and Table 1. The different treatments were evaluated using the entropy weight method. The subjective weights were assigned according to the importance of the indicators under the local conditions scored by 16 experts (4 in agricultural economy, 4 in soil ecology, 4 in plant nutrition, and 4 in agricultural water conservancy). As shown in Table 2, both the subjective and the objective weights tended to emphasize the importance of tomato yield and soil salt content in the plough layer. This was mainly due to the fact that high yield and high benefit were still the main targets for agriculture in our experimental area, whilst excessive soil salinity was a key factor limiting the yield formation and was a serious soil environmental problem that needed to be solved urgently.

**Figure 3:**
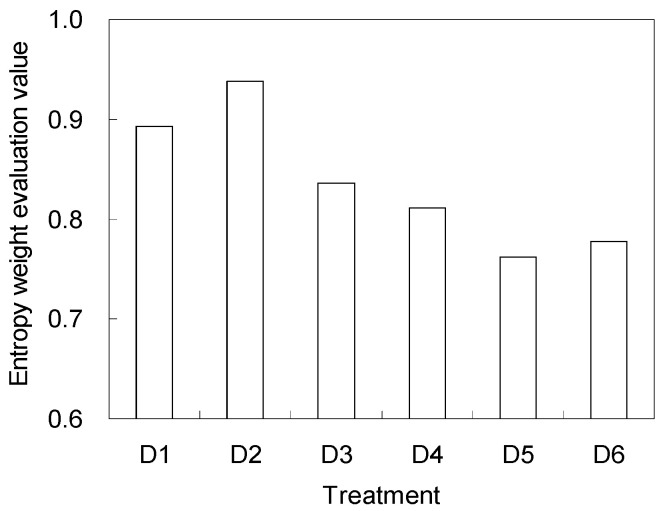
Entropy weight evaluation value for the different treatments (D1, D2, D3, D4, D5, and D6 represent 30, 45, 60, 75, 90, and 105 cm buried depths of drainage pipes, respectively).

The evaluation results of the entropy weight method for the different treatments are shown in Figure 3. According to the principle of the method, D2 (45 cm) was considered as the most suitable buried depth with the optimal comprehensive benefit when considering soil salt, available nitrogen, yield, sugar to acid ratio, as well as water and nitrogen use efficiency.

## 4. Discussion

A deeper buried depth of drainage pipes resulted in higher available nitrogen concentration in plough layer, possibly because the surface soluble nitrogen could not reach the position of the buried pipe and then flowed away with irrigation water, and it would have to return to the surface soil with the water by evaporation. It is generally believed that there is a significant positive correlation between crop yield and available nitrogen content [16,17,18]. Interestingly, although D6 treatment possessed the highest soil available nitrogen content, the tomato yield was not in the highest level, which might be on account of higher salt content which restricted crop uptake of nitrogen (Figure 2a).

The tomato yield was not directly related to the buried depth of drainage pipes, which might be related to the various factors affected by subsurface drainage. Early results showed that the yield was associated with soil salinity, water distribution, available nutrient contents, etc. [1,19,20]. The sugar to acid ratio of the tomato increased as the buried depth increased, which might be caused by the high soil salinity under deeper drainage (Figure 2a), and the high salinity could promote the accumulation of sugar in fruit. The mechanism was that the activity of invertase increased under salt stress, resulting in the increase of soluble sugar concentration in tomato fruit [21]. Nitrogen loss caused by shallower buried depth of drainage pipes might be responsible for lower nitrogen use efficiency (Table 1). Therefore, additional nitrogen fertilizer should be applied if the drainage pipe is buried shallowly.

Various indexes should be taken into consideration when developing agricultural management schemes. To avoid the adverse effects of subjective experience on decision-making results, it is necessary to use statistical models, such as the early study by Shao [22], which used the projection pursuit classification model to select the optimal drip irrigation scheme. Our previous research also used the projection pursuit model to optimize the irrigation and drainage scheme for greenhouse tomato [10]. Yan Jianwen [9] used principal component analysis method to optimize the water and nitrogen cooperation scheme for flue-cured tobacco. At present, the entropy weight coefficient evaluation model has been well used in developing the subsurface drainage scheme. In comprehensive consideration of the crop yield, quality, and irrigation amount, Shao [23] employed the entropy weight model to optimize the subsurface drainage layout scheme under rain-shelter cultivation in southern China. The entropy weight method has now played a positive role in the decision-making schemes with multi-objectives and multi-indexes in agriculture [24].

In our study, we aimed at the shallow groundwater level and the high soil salinity in greenhouses of South China and carried out an experiment of subsurface drainage. Since our early study has found a suitable buried space between drainage pipes [25], the main purpose of this study was to determine the buried depth of drainage pipes. The indexes selected by this study were tomato marketable yield, sugar to acid ratio, nitrogen use efficiency, water use efficiency, salt reduction efficiency, and available nitrogen loss rate, since the application of the subsurface drainage was expected to achieve high crop yield and quality, improve soil condition, increase resource use efficiency, and promote the sustainable development of greenhouse agriculture. According to the calculation results of objective and subjective weight, yield, and soil salinity were two of the most important indexes. The results of the entropy weight model showed that 45 cm was the optimal buried depth in our study, followed by 30 cm. However, considering that 30 cm was not convenient for the field tillage of plough machines, the buried depth of 60 cm was recommended by the entropy weight method as the third choice. Therefore, we recommend the buried depth of drainage pipes to be in the range of 45–60 cm.

The deficiency in our study was that we focused on the soil in the plough layer whilst ignoring the indicators in the profile layer. This will limit deeper development of mechanisms. In similar studies, indicators such as soil thickness, grain size, composition, density, effective porosity, coefficient of permeability, were and should be observed.

Moreover, from the previous research results, a greater number of samples and indicators were of great importance to improve the accuracy of the calculation results. The statistical technology, such as the entropy weight method has broad prospects in optimization of agricultural management schemes. In future, the study on the construction of index systems and the evaluation of model calculation reliability should be further strengthened.

## 5. Conclusions

Both objective and subjective weights indicated that tomato yield and soil electrical conductivity were relatively more important than other indexes, and their comprehensive weights were 0.43 and 0.34, respectively. The buried depth which possessed the optimal comprehensive benefits was 45 cm, with an entropy evaluation value of 0.94. Under the 45 cm buried depth, the loss rate of available nitrogen was 13.9%, the decrease rate of soil salt was 49.2%, and after one-season of cultivation, the tomato yield, sugar to acid ratio, nitrogen use efficiency, and water use efficiency were 112 kg·ha^-1^, 8.3, 39.7%, and 42.0%, respectively. In consideration of the economic benefits, ecological benefits, and maneuverability, we recommend the buried depth of drainage pipes to be in a range of 45–60 cm in the south of China.

## Figures and Tables

**Table 1:** The tomato marketable yield, sugar to acid ratio, nitrogen use efficiency, and water use efficiency under different treatments.

Treatment	Yield (kg/ha)	Sugar to Acid Ratio	Nitrogen Use Efficiency (%)	Water Use Efficiency (%)
D1	102.3 ± 2.05 b	7.73 ± 0.41 d	35.6 ± 3.01 d	45.0 ± 2.13 a
D2	112.0 ± 5.10 a	8.30 ± 0.24 cd	39.7 ± 2.16 cd	42.0 ± 1.63 ab
D3	86.3 ± 3.68 d	8.33 ± 0.25 cd	42.1 ± 1.49 bc	39.9 ± 1.28 b
D4	98.0 ± 4.55 bc	8.93 ± 0.53 bc	43.4 ± 2.56 bc	39.0 ± 1.59 b
D5	87.0 ± 3.27 d	9.40 ± 0.33 ab	46.7 ± 3.43 ab	38.9 ± 1.88 b
D6	92.7 ± 3.68 cd	9.90 ± 0.33 a	49.5 ± 2.58 a	34.3 ± 1.27 c

Note: Means followed by the same letter (a, b, c, d) do not differ significantly at a 0.05 level, according to Duncan’s multiple range test. D1, D2, D3, D4, D5 and D6 represent 30, 45, 60, 75, 90, and 105 cm buried depths of drainage pipes, respectively.

**Table 2:** The subjective and objective weight for the indicators.

Weight	Yield	Sugar to Acid Ratio	Nitrogen Use Efficiency	Water Use Efficiency	Soil Salt	Available Nitrogen
Objective weight	0.23	0.11	0.18	0.11	0.34	0.05
Subjective weight	0.37	0.17	0.06	0.13	0.20	0.07
Comprehensive weight	0.43	0.09	0.05	0.07	0.34	0.02
